# The Role of Language in the Hearing-Cognition Association: Hispanic Community Health Study/Study of Latinos

**DOI:** 10.1093/geroni/igaf122.3425

**Published:** 2025-12-31

**Authors:** Pablo Martinez-Amezcua, Xi Wang, Laura Coco, Ariana Stickel, Hector Gonzalez, Linda Gallo, Daniela Sotres-Alvarez

**Affiliations:** Johns Hopkins Bloomberg School of Public Health, Baltimore, Maryland, United States; Johns Hopkins University Bloomberg School of Public Health, Baltimore, Maryland, United States; San Diego State University, San Diego, California, United States; San Diego State University, San Diego, California, United States; University of California, San Diego, San Diego, California, United States; San Diego State University, San Diego, California, United States; The University of North Carolina at Chapel Hill, Chapel Hill, North Carolina, United States

## Abstract

**Background:**

Hearing loss (HL) is linked to lower cognitive function, yet most research has focused on non-Hispanic/Latino White adults and English speakers. In 2020, 60 million Hispanic/Latino individuals lived in the US, including 16 million with limited English proficiency. Lower English acculturation—a proxy for proficiency—is associated with poorer cognitive performance, but its role in moderating the HL–cognition relationship remains unclear.

**Methods:**

We analyzed cross-sectional baseline data from 8,089 Hispanic/Latino adults aged 45 to 74 (mean age = 55; 61% female; 86% Spanish speakers) from the multi-site Hispanic Community Health Study/Study of Latinos cohort. HL was defined as a better-ear pure tone average >25 dB. Language acculturation was assessed using the language domain of the Short Acculturation Scale for Hispanics and categorized into tertiles. We derived global cognitive function scores (z-transformed) from a neurocognitive battery. We used survey-weighted regression models, adjusted for demographics and health factors, to examine the interaction between HL and acculturation tertiles on cognitive function.

**Results:**

Among individuals with high acculturation, those with HL (prevalence 14%) had a 0.045‐unit lower cognitive score than those without. HL and cognition associations were stronger for those with medium and low acculturation (0.097 and 0.099 units lower cognitive scores, respectively). However, the differences in the HL coefficients across acculturation levels were not significant (β HL* medium = –0.052 and β HL* low = –0.054, p > 0.05 for both).

**Conclusions:**

Among Hispanic/Latino adults, HL is associated with lower cognitive function. The heterogeneity in this association across acculturation levels was not statistically significant.

